# Brain metastasis in basaloid undifferentiated anal carcinoma: A case report

**DOI:** 10.3892/ol.2014.1845

**Published:** 2014-01-31

**Authors:** JORGE HERNANDO-CUBERO, VICENTE ALONSO-ORDUÑA, ALBA HERNANDEZ-GARCIA, ANA CEBOLLERO DE MIGUEL, NATALIA ALVAREZ-GARCIA, ANTONIO ANTON-TORRES

**Affiliations:** 1 Department of Medical Oncology, Miguel Servet University Hospital, Zaragoza 50009, Spain; 2 Department of Surgery, Miguel Servet University Hospital, Zaragoza 50009, Spain

**Keywords:** anal cancer, basaloid carcinoma, cloacogenic carcinoma, brain metastasis

## Abstract

Anal cancer is a rare tumor that accounts for 2% of all colorectal neoplasms. The brain is a rarely affected organ. The aim of the present study was to the review the only four cases of anal cancer brain metastases previously published in the literature. In addition, the current study presents the case of a 69-year-old male diagnosed with basaloid undifferentiated carcinoma of the anal canal (stage IV with liver, lung and bone metastasis). Despite the patient’s good response to chemotherapy and the achievement of a partial response that was maintained for 14 months, brain metastases developed. Although radiotherapy was administered, the patient succumbed to the condition 12 weeks after the diagnosis of brain metastasis.

## Introduction

Anal cancer is a rare tumor among gastrointestinal tract neoplasms. Squamous cell carcinomas are the most common subtype. The most common subtype is squamous cell carcinoma. Basaloid squamous cell carcinoma is a rare and aggressive variant of squamous cell carcinoma that normally arises in the upper aerodigestive tract and less frequently in the lungs, anus, vagina and uterine cervix (1).

Overall, ≤20% of patients present with disseminated disease at the time of diagnosis. Brain metastasis is a rare entity, with only a total of four cases have been previously reported in the literature. To the best of our knowledge, this case report presents the fifth case of anal carcinoma with brain metastasis.

## Case report

A 69-year-old male presented with a six-month history of anorectal pain, tenesmus and an anal tumor. In addition, the patient complained of back pain, fatigue and hyporexia. The patient’s medical history included glaucoma and cataracts, and an 80 pack-year smoking history. A physical examination showed hepatomegaly, a painful mass in the external anal sphincter and a performance status of 1.

Colonoscopy found an ulcerated mass within 6 cm of the anal verge. Pathological specimens were obtained during the colonoscopy procedure and the histological report showed a basaloid undifferentiated carcinoma. Staging computed tomography (CT) scans of the chest, abdomen and pelvis exhibited mesorectal lymphadenopathy and multiple pulmonary and liver metastases. Due to the back pain, a bone scintigraphy was also performed and uptake was revealed at the coccix, right sacral ala and pubis.

**Figure 1:**
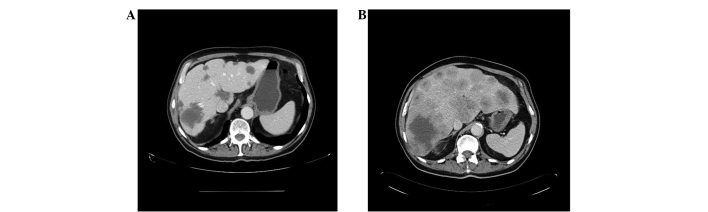
Liver metastasis (A) following and (B) prior to chemotherapy.

The patient was diagnosed with basaloid undifferentiated carcinoma of the anal canal, stage IV (T3N2M1), and underwent treatment in October 2011. The patient was treated with cisplatin (75 mg/m^2^) and 5-fluorouracil (5FU; 750 mg/m^2^/day as a continuous infusion for five days). An evaluation following the third and sixth cycle of treatment recorded a partial response (Fig. 1). Toxicity included asthenia G2, nausea G1 and peripheral neuropathy G1.

**Figure 2:**
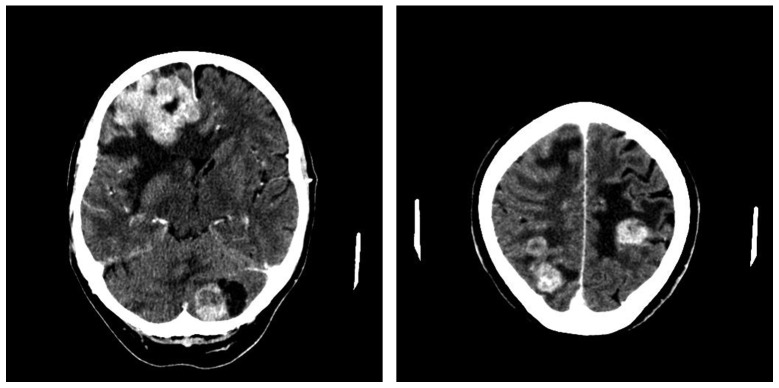
Brain metastasis at the time of diagnosis.

Subsequent to 14 months of follow-up after treatment with chemotherapy, the patient developed neurological symptoms consisting of loss of strength in the left arm that evolved to self-limited episodes of myoclonic status, which became generalized. Cranial CT scans found multiple lesions indicative of metastases in the right parietal, left rolandic, cortical and subcortical, cerebellar and frontal lobes, with tonsillar herniation (Fig. 2). Steroid therapy was initiated with prompt symptomatic improvement and cranial radiotherapy was planned and delivered (30 Gy).

Despite treatment, the general condition of the patient gradually deteriorated until they succumbed 12 weeks after the diagnosis of brain metastasis.

## Discussion

Anal cancer (including the anus, anal canal or anorectum) accounts for 2.2% of all gastrointestinal malignancies (2). The disease usually occurs in the sixth decade of life. Although relatively rare, the incidence of anal cancer is increasing due to its risk factors (3), such as anal-genital human papillomavirus (HPV) infection, immunosuppression associated with human immunodeficiency virus or transplantation and smoking (4,5). HPV infection is associated with 97% of anal cancers (6).

These tumors are divided into two categories, tumors of the anal canal or anal margin, based on their anatomical location. The majority of anal canal tumors are squamous cell carcinomas (7). Although the tumors have been traditionally divided into well-differentiated keratinizing and non-keratinizing (cloacogenic/basaloid and transitional) types, the two types have a similar natural history. Therefore, no distinction has been determined in their management (8). Other rarer types include adenocarcinoma, anaplastic carcinoma, undifferentiated tumors and melanomas (7).

Squamous cell carcinoma also predominates in the anal margin subtype, which is usually well-differentiated and keratinizing (9). Overall, squamous carcinomas account for 75% of anal cancers (10).

Clinically presenting with rectal bleeding (45%) and anorectal pain or urgency (30%) (10), 10–20% of patients exhibit extrapelvic disseminated disease at the time of diagnosis. The most important organ for metastases is the liver (12). The five-year survival rate at stage IV is 20.9% in squamous cell carcinomas and 7.4% in non-squamous cell carcinomas (8). Cisplatin-based chemotherapy in combination with 5FU and paclitaxel is the standard treatment of metastatic anal carcinoma (13), with radiotherapy provided for local treatment (14). The presence of epidermal growth factor receptor (EGFR) overexpression in anal carcinoma indicates a role for anti-EGFR therapies, such as cetuximab (15).

A total of four cases of anal cancer with brain metastasis have been previously reported in the literature. The first was identified in a review of 373 cases of anorectal ‘transitional cloacogenic carcinomas’ (now considered squamous cell carcinomas) by Klotz *et al* (1967), however, no further data on this case is available (16). The following three cases were published in 1991, 2011 and 2012, respectively (Table I).

Davidson and Yong reported the case of a 61-year-old female with a single brain lesion appearing eight years after abdominoperineal amputation of non-metastatic anal carcinoma at diagnosis. The patient was treated with surgery and adjuvant radiotherapy with posterior recurrence of the primary tumor (17).

Rughani *et al* (2011) reported the case of a 63-year-old patient with liver metastasis at diagnosis who received chemoradiation treatment. Following treatment, the patient showed prompt neurological symptoms and isolated brain metastasis was identified. The patient underwent surgery and radiation therapy, but succumbed 14 weeks later (10).

Finally, Austin Gassman *et al* recently reported the case of a 67-year-old patient with a single liver metastasis at diagnosis, who received neoadjuvant chemoradiation therapy and subsequently underwent surgery for the primary tumor and liver metastasis. The patient presented with blurred vision and right ptosis three months after surgery. A tissue sample confirmed brain metastasis. Radiotherapy was planned but was not performed as the patient succumbed to the disease (18).

The present case had the peculiarity of being a basaloid undifferentiated carcinoma, unlike the previous cases which were the squamous type (although poorly differentiated). The patient was diagnosed with metastatic disease similar to the majority of the cases described previously. The present case had a good response to the initial treatment with chemotherapy, achieving a partial response maintained for 14 months. Due to the appearance of neurological symptoms, a cranial CT scan was performed, which revealed the first documented case of brain and cerebellar metastasis for this condition. Despite receiving radiation therapy, the patient clinically deteriorated until the patient succumbed 12 weeks following the diagnosis of brain metastasis.

Despite its rarity, brain metastasis must be considered in any patient with anal cancer and neurological symptoms. All five reported cases have been squamous carcinomas with disseminated disease at diagnosis, with the exception of one case. In addition, brain metastasis has been associated with a poor prognosis. Radiation therapy may be administered with palliative intent, alone or in combination with surgery in selected cases.

## Figures and Tables

**Table I:** Comparison of the only cases of brain metastasis published in the literature to date.

First author/s (ref)	Age, years	Gender	Histology	Stage at diagnosis	Distant metastasis	Initial treatment	Months to cerebral metastasis	Survival after brain metastasis diagnosis, weeks	Brain metastasis treatment
Klotz *et al* (15)	NA	NA	Cloacogenic (squamous)	IV	No	NA	NA	NA	NA
Davidson and Yong (16)	61	Female	Basaloid (squamous)	II	No	Surgery	96	NA	Surgery and RT
Rughani *et al* (9)	63	Female	Squamous	IV	Liver	CT and RT	NA	14	Surgery
Gassman *et al* (17)	67	Male	Squamous undifferentiated	IV	Liver	CT, RT and surgery	6	NA	RT
Present case	69	Male	Basaloid (squamous) undifferentiated	IV	Liver, lung and bone	CT	14	12	RT

NA, not available; CT, chemotherapy; RT, radiotherapy.
